# Data on the histological and immune cell response in the popliteal lymph node in mice following exposure to metal particles and ions

**DOI:** 10.1016/j.dib.2016.08.037

**Published:** 2016-08-27

**Authors:** Bethany Winans, Brooke E. Tvermoes, Kenneth M. Unice, Michael Kovochich, Ernest S. Fung, Whitney V. Christian, Ellen Donovan, Brent L. Finley, Ian Kimber, Dennis J. Paustenbach

**Affiliations:** a Cardno ChemRisk, LLC.; 101 2nd Street, Suite 700, San Francisco, CA 94105, USA; b Cardno ChemRisk, LLC.; 4940 Pearl East Circle, Suite 100, Boulder, CO 80301, USA; c Cardno ChemRisk, LLC.; 20 Stanwix Street, Suite 505, Pittsburgh, PA 15222, USA; d Cardno ChemRisk, LLC.; 130 Vantis Drive, Suite 170, Aliso Viejo, CA 92656, USA; e University of Manchester, Faculty of Life Sciences, Oxford Road, Manchester M13 9PT, UK

**Keywords:** Metals, Particles, Hypersensitivity, Lymph node cell activation, Immune stimulation, Metal-on-metal implants, Popliteal lymph node assay

## Abstract

Hip implants containing cobalt–chromium (CoCr) have been used for over 80 years. In patients with metal-on-metal (MoM) hip implants, it has been suggested that wear debris particles may contribute to metal sensitization in some individuals, leading to adverse reactions. This article presents data from a study in which the popliteal lymph node assay (PLNA) was used to assess immune responses in mice treated with chromium-oxide (Cr_2_O_3_) particles, metal salts (CoCl_2_, CrCl_3_, and NiCl_2_) or Cr_2_O_3_ particles with metal salts (“A preliminary evaluation of immune stimulation following exposure to metal particles and ions using the mouse popliteal lymph node assay” (B.E. Tvermoes, K.M. Unice, B. Winans, M. Kovochich, E.S. Fung, W.V. Christian, E. Donovan, B.L. Finley, B.L. Kimber, I. Kimber, D.J. Paustenbach, 2016) [1]). Data are presented on (1) the chemical characterization of TiO_2_ particles (used as a particle control), (2) clinical observations in mice treated with Cr_2_O_3_ particles, metal salts or Cr_2_O_3_ particles with metal salts, (3) PLN weight and weight index (WI) in mice treated with Cr_2_O_3_ particles, metal salts or Cr_2_O_3_ particles with metal salts, (4) histological changes in PLNs of mice treated with Cr_2_O_3_ particles, metal salts or Cr_2_O_3_ particles with metal salts, (5) percentages of immune cells in the PLNs of mice treated with Cr_2_O_3_ particles, metal salts or Cr_2_O_3_ particles with metal salts, and (6) percentages of proliferating cells in the PLNs of mice treated with Cr_2_O_3_ particles, metal salts or Cr_2_O_3_ particles with metal salts.

**Specifications Table**

**Table:** 

Subject area	*Biology*
More specific subject area	*Toxicology, Popliteal Lymph Node Assay, Metal-on-Metal Hip Implants, Histology, Metal Sensitization, Immune Stimulation*
Type of data	*Tables, figures*
How data was acquired	*Observation, Microscope and Hemocytometer, Scanning Electron Microscope (Hitachi S5500), Electron Dispersive Spectroscope, Flow Cytometer (BD FacsScan)*
Data format	*Analyzed*
Experimental factors	*BALB/c mice were given a single footpad injection of Cr*_*2*_*O*_*3*_*particles, metal salts (CoCl*_*2*_*, CrCl*_*3*_*, and NiCl*_*2*_*), Cr*_*2*_*O*_*3*_*particles plus metal salts, or controls. Four to 11 days later the immune response in the popliteal lymph node (PLN) was assessed.*
Experimental features	*Four to 11 days following the footpad injection of Cr*_*2*_*O*_*3*_*particles, metal salts (CoCl*_*2*_*, CrCl*_*3*_*, and NiCl*_*2*_*), Cr*_*2*_*O*_*3*_*particles plus metal salts, or controls, clinical observations and the weight and weight index of the PLN were assessed. Flow cytometry was performed to evaluate the proportion of various immune cells and proliferation of cells in the PLN following treatment with the test articles. Additionally, histology was performed on the PLNs of treated mice.*
Data source location	*MB Labs, Spinnerstown, PA; Calvert Labs, Scott Township, PA; and RJ Lee Group, Monroeville, PA*
Data accessibility	*With this article*

**Value of the data**

• These data represent the first use of the PLNA to test the immune response in mice treated with Cr_2_O_3_ particles, and will be of value to researchers studying metal sensitization.
• Histological parameters, including development of germinal centers and hyperplasia of lymphocytes in the cortex, are presented for mice treated with the test articles. These data will be of value in trying to understand the type of immune response observed following treatment with metal particles and ions and to researchers evaluating PLN histology following treatment with metals or other agents.
• Flow cytometry was performed, evaluating the response of various types of immune cells, including B220^+^, CD3^+^, CD4^+^, CD8^+^, I-AD^+^ and CD69^+^ cells. These data will be of value in trying to fully characterize the type of immune response observed following treatment with metal particles and ions and to researchers evaluating changes in the percentage of immune cells in the PLN following treatment with metals or other agents.
• PLN weight, flow cytometry and histology endpoints were all evaluated in the same treatment groups, allowing comparison across multiple endpoints to better assess the immune response. These data will be of value to researchers evaluating the immune response following treatment with metals or other agents.

## 1 Data

**Fig. 1:**
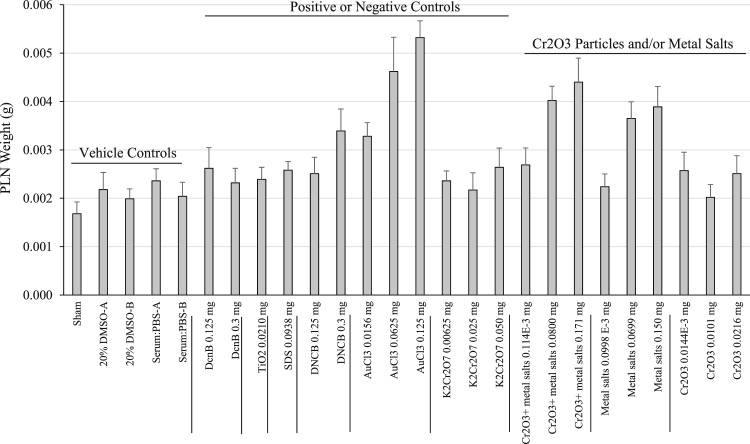
PLN weight four days following footpad injection in Experiment 1. Mice either received no injection (sham), or were injected with a vehicle control or test article as indicated. Four days after treatment, mice were sacrificed and the ipsilateral PLN was weighed. Data are presented as the mean+SE.

**Fig. 2:**
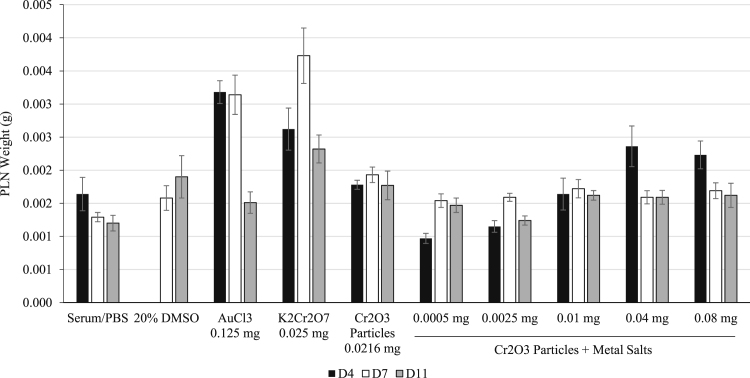
PLN weight 4, 7 and 11 days following footpad injection in Experiment 2. Mice were injected with a vehicle control or test articles as indicated. On D4, D7, and D11, mice were sacrificed and the ipsilateral PLN was weighed. Note that no D4 data were obtained for the 20% DMSO treatment group. Data are presented as the mean±SE.

**Fig. 3:**
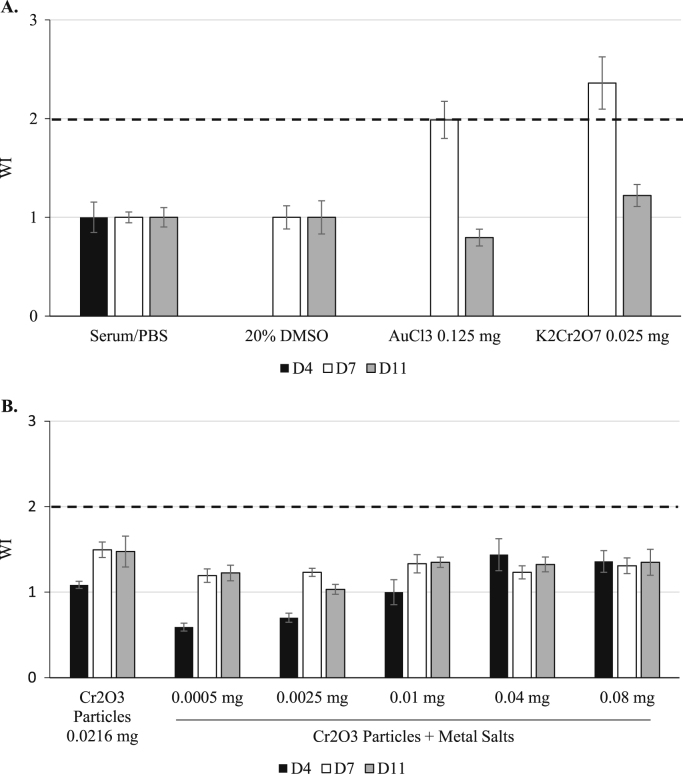
Weight index (WI) following footpad injections in Experiment 2. Mice were injected with vehicle controls or positive controls (A) or test articles (B), as indicated. On D4, D7 and D11, PLN weight was recorded. The WI was calculated as follows: PLN weight _test animal_ / average PLN weight _appropriate vehicle control_. Note that no D4 data were obtained for the 20% DMSO treatment group; therefore, WI values were not calculated for AuCl_3_ and K_2_Cr_2_O_7_ at D4. The dashed line indicates a WI value of 2, which is the threshold value for a positive response in the PLNA. Data are presented as the mean±SE.

This data in brief article contains data on the induced immune response in the popliteal lymph node (PLN) of mice treated with Cr_2_O_3_ particles and/or metal salts from two experiments. From the first experiment, the following data are presented: compositional analysis of TiO_2_ particles (Supplementary Fig. 1); evaluation of localized inflammation (Supplementary Table 1) and discoloration (Supplementary Table 2) in the footpad; mean footpad swelling (Supplementary Fig. 2); mean change in body weight (Supplementary Fig. 3); mean PLN weight (Fig. 1); histological evaluation of the PLN (Table 1, Table 2, Table 3, Table 4); and representative flow cytometry plots for the percentage of cells positive for CD3 and B220 (Supplementary Fig. 4, 5), I-A^D^ and CD69 (Supplementary Fig. 6, 7), CD4 and CD8 (Supplementary Fig. 8, 9) and BrdU (Supplementary Fig. 10, 11). From the second experiment, the following data are presented: evaluation of localized inflammation (Supplementary Table 3) and discoloration (Supplementary Table 4) in the footpad; mean footpad swelling (Supplementary Fig. 12); mean change in body weight (Supplementary Fig. 13); and mean PLN weight (Fig. 2) and WI (Fig. 3). Please refer to [1] for related data and interpretations.

## 2 Experimental design, materials and methods

The materials and methods have been described previously [1]. Briefly, the materials and methods were as follows:

### 2.1 Animals

Nulliparous, experimentally naïve, 6–8 week old female BALB/c mice (Charles River Laboratories) were housed in metal-free, disposable plastic cages. The mice were maintained on a 12-hour light/dark cycle in a temperature-controlled environment, and were acclimatized for at least five days. Distilled water and rodent chow were provided *ad libitum*. All procedures complied with acceptable standards of animal welfare and humane care by the Institutional Animal Care and Use Committee (IACUC) of MB Research (Spinnerstown, PA) and Calvert Labs (Scott Township, PA).

### 2.2 Chemicals and reagents

The following reagents were purchased from the source listed in Table 5.

### 2.3 Characterization of metallic particles

The morphology of the TiO_2_ particles was determined using a Hitachi S5500 Ultra-high Resolution Scanning Electron Microscope at an accelerating voltage of 2.0 kV with secondary electron contrast at RJ Lee Group (Monroeville, PA). Composition of the TiO_2_ particles was determined using a Bruker energy dispersive spectroscopy (EDS) detector at an accelerating voltage of 20 kV.

### 2.4 PLNA

Mice were anesthetized with isoflurane and injected subcutaneously with 50 μL of vehicle or test article into the right hind footpad. The dosing groups are presented in Table 6.

All dilutions were prepared fresh daily and were stirred or vortexed until homogeneous. Dilutions were vortexed prior to dosing each mouse. Treatment doses were based on previous literature and dose-range finding studies [2], [3], [4]. See [1] for a detailed description of the rationale of dose formulations.

### 2.5 Assessment of footpad swelling and general toxicity

The injection site of all animals was evaluated for signs of swelling or discoloration, and animals were evaluated for distress or signs of general toxicity approximately four hours after injection and once daily until sacrifice. Right hind footpad swelling was measured at 1, 2 and 4 days post injection in Experiment 1 and from Day 0 to 11 in Experiment 2 using a digital micrometer. Body weights were measured immediately prior to treatment on Day 0 and at sacrifice on Day 4, 7 or 11. Results for footpad swelling and percent of initial body weight are expressed as mean±standard error (SE).

### 2.6 PLN weight and cell proliferation

In Experiment 1, five hours before sacrifice on Day 4, mice were administered bromodeoxyuridine (BrdU) dissolved in PBS (3 mg per mouse; intraperitoneal). In Experiment 2, no BrdU was administered. Mice were euthanized with carbon dioxide inhalation, and the ipsilateral PLNs were excised from each mouse. PLNs were placed in PBS, adherent fatty tissue was removed, and PLNs were weighed. Results for ipsilateral PLN weight and weight index (Experiment 2 only) are expressed as mean±standard error (SE).

For each individual PLN, a single cell suspension was made by gentle disaggregation with a disposable pestle, and cells were centrifuged, washed in PBS, and re-suspended in RPMI. In Experiment 1, the isolated LNCs were used for either determination of BrdU incorporation (fixed in 75% EtOH and stored up to one week at −20 °C) or for flow cytometric analyses (stored overnight at 2–8 °C).

To determine BrdU incorporation in Experiment 1, cells were denatured with HCl Triton X Buffer (1 N HCl, 0.5% Triton X) and neutralized by washing with borate buffer (pH 8.5). Nuclei were washed with a staining buffer, incubated with BrdU-FITC (BD Biosciences, clone B44), washed again with staining buffer and resuspended in PBS containing RNase A (Fisher Scientific) and propidium iodide (PI, Sigma-Aldrich). Samples were incubated at room temperature for 30 min, and the percentage of BrdU^+^ nuclei (i.e., percentage of proliferating lymphocytes in the PLN) was determined with a BD FacScan^®^ flow cytometer.

### 2.7 Flow cytometry

In Experiment 1, PLN cells were incubated with either Rat IgG (for B220, CD3, CD4, and CD8) or hamster/mouse IgG (for I-AD and CD69) for ten minutes to block non-specific binding. Approximately 5x10^5^ cells were incubated for 30–45 min on ice with fluorescently-conjugated antibodies in the following pairs: (1) B220-FITC (BD Pharmingen, clone RA3-6B2) and CD3-PE (BD Pharmingen, clone 17A2); (2) CD4-PE (BD Pharmingen, clone RM4-5) and CD8-FITC (BD Pharmingen, clone 53-6.7); or (3) I-AD-FITC (Acris Antibodies, clone 34-5-3S) and CD69-PE (BD Pharmingen, clone H1.2F3). Cells were fixed with 70% ethanol and analyzed by flow cytometry on a BD FacScan^®^ flow cytometer using 15 mW of power at 488 nm excitation wavelength. Data was acquired on BD CellQuest version 3.3 acquisition software, and CellQuest™ and FlowJo were used for data analysis.

### 2.8 Histology

For histological evaluation in Experiment 1 (*n*=2 per treatment group), PLNs were fixed in 10% neutral-buffered formalin and embedded in paraffin. Tissues were cut to 5 μm thickness and stained with hematoxylin and eosin (H&E). The pathologist evaluated the following parameters on a 0 to 4 scale: (1) number of primary follicles; (2) number of secondary follicles/germinal centers; (3) maturity of lymphocytes in the cortex; (4) lymphocyte hyperplasia in the paracortex; (5) plasma cells in the medullar cords; (6) necrosis; (7) acute inflammation (edema); and (8) acute inflammation (infiltration of polymorphonuclear cells). The vehicle control groups were used to establish the baseline scores, and the remaining groups were evaluated blinded as to treatment group.

## Figures and Tables

**Table 1:** Histology of the PLN of sham control and vehicle controls in Experiment 1. Four days following sham injection or injection of vehicle controls, the indicated histological parameters were evaluated in the PLN.

**Parameter**	**Treatment Group**									
	**Sham**		**20% DMSO-A**		**20% DMSO-B**		**Serum:PBS-A**		**Serum:PBS-B**	
	**Animal #**									
	**389**	**390**	**259**	**260**	**399**	**400**	**319**	**320**	**469**	**470**
Number of secondary follicles/germinal centers^a^	−	−	−	−	−	NA^b^	−	−	−	−
Number of primary follicles^c^	++++	++	++	+++	++	NA	++	++	++	++
Maturity of lymphocytes in cortex^d^	+	+	+	+	+	NA	+	+	+	+
Lymphocyte hyperplasia in paracortex^e^	++	+	+	+	+	NA	+	+	−	+
Presence of plasma cells in medullary cords^f^	−	−	−	−	−	NA	−	−	−	−
Necrosis^g^	−	−	−	−	−	NA	−	−	−	−
Acute inflammation: Edema^h^	−	−	−	−	−	NA	−	−	−	−
Acute inflammation: Polymorphonuclear cells^i^	−	−	−	−	−	NA	−	−	−	−

a : − = absent, + =1–2 follicles, ++ =3–4 follicles.

b : NA=tissue not available for analysis.

c : − = no cortex present on sample, + =1-2 follicles, ++ =3-4 follicles, +++ =5-6 follicles, ++++ =7-8 follicles.

d : + =all small lymphocytes, ++ =~5% immature lymphocytes, +++ =~10% immature lymphocytes.

e : − = absent, + =minimal size, mature lymphocytes, ++ =medium size, ~5% immature lymphocytes, +++ =large size, ~10% immature lymphocytes, ++++ =extra-large size, ~20% immature lymphocytes.cords filled.

f : − = none observed, + =several, ++ =readily apparent, +++ =cords filled.

g :−= absent, + =minimal foci.

h :− = absent, + = minimal foci.

i : − = absent, + = minimal foci.

**Table 2:** Histology of the PLN of positive and negative chemical controls in Experiment 1. Four days following sham injection or injection of vehicle controls, the indicated histological parameters were evaluated in the PLN.

**Parameter**	**Treatment Group**									
	**DNCB 0.125** **mg**		**DNCB 0.3** **mg**		**SDS 0.0938** **mg**		**DCNB 0.125** **mg**		**DCNB 0.3** **mg**	
	**Animal #**									
	**409**	**410**	**279**	**280**	**269**	**270**	**419**	**420**	**429**	**430**
Number of secondary follicles/germinal centers^a^	++	−	−	−	−	−	+	+	−	−
Number of primary follicles^b^	+++	++	+	+++	++	++	+	+++	++	++
Maturity of lymphocytes in cortex^c^	++	+	+	+	+	+	+	++	+	+
Lymphocyte hyperplasia in paracortex^d^	+	++	−	++	−	+	+	+	−	++
Presence of plasma cells in medullary cords^e^	−	−	−	−	−	−	−	−	−	−
Necrosis^f^	−	−	−	−	−	−	−	−	−	−
Acute inflammation: Edema^g^	−	−	−	−	−	−	−	−	−	−
Acute inflammation: Polymorphonuclear cells^h^	−	−	−	−	−	−	−	+	−	−

a : − = absent, + =1–2 follicles, ++ =3–4 follicles.

b : − = no cortex present on sample, + =1–2 follicles, ++ =3–4 follicles, +++ =5–6 follicles, ++++ =7–8 follicles.

c : + =all small lymphocytes, ++ =~5% immature lymphocytes, +++ =~10% immature lymphocytes.

d : − = absent, + =minimal size, mature lymphocytes, ++ =medium size, ~5% immature lymphocytes, +++ =large size, ~10% immature lymphocytes, ++++ =extra-large size, ~20% immature lymphocytes.

e : − = none observed, + =several, ++ =readily apparent, +++ =cords filled.

f :− = absent, + =minimal foci.

g : − = absent, + = minimal foci.

h : − = absent, + = minimal foci.

**Table 3:** Histology of the PLN of positive and negative metal controls in Experiment 1. Four days following sham injection or injection of vehicle controls, the indicated histological parameters were evaluated in the PLN.

**Parameter**	**Treatment Group**													
	**AuCl**_**3**_**0.0156** **mg**		**AuCl**_**3**_**0.0625** **mg**		**AuCl**_**3**_**0.125** **mg**		**K**_**2**_**Cr**_**2**_**O**_**7**_**0.00625** **mg**		**K**_**2**_**Cr**_**2**_**O**_**7**_**0.025** **mg**		**K**_**2**_**Cr**_**2**_**O**_**7**_**0.050** **mg**		**TiO**_**2**_**0.0210** **mg**	
	**Animal #**													
	**439**	**440**	**449**	**450**	**459**	**460**	**289**	**290**	**299**	**300**	**309**	**310**	**479**	**480**
Number of secondary follicles/germinal centers^a^	−	−	−	−	−	+	−	−	NA^b^	NA	−	−	−	−
Number of primary follicles^c^	++	++	++	++	+++	++	−	++	NA	NA	++	+++	+++	++
Maturity of lymphocytes in cortex^d^	+	+	+	+++	+	++	−	+	NA	NA	+	+	++	+
Lymphocyte hyperplasia in paracortex^e^	+	+	++	++	++	++	−	+	NA	NA	++++	++++	+	−
Presence of plasma cells in medullary cords^f^	−	−	−	−	−	−	−	−	NA	NA	−	−	−	−
Necrosis^g^	−	−	−	−	−	−	−	−	NA	NA	−	−	−	−
Acute inflammation: Edema^h^	−	−	−	−	−	−	−	−	NA	NA	−	−	−	−
Acute inflammation: Polymorphonuclear cells^i^	−	−	−	−	−	−	−	−	NA	NA	−	−	−	−

a : − = absent, + =1–2 follicles, ++ =3–4 follicles.

b : NA=tissue not available for analysis.

c : − = no cortex present on sample, + =1-2 follicles, ++ =3-4 follicles, +++ =5-6 follicles, ++++ =7-8 follicles.

d : + =all small lymphocytes, ++ =~5% immature lymphocytes, +++ =~10% immature lymphocytes.

e : − = absent, + =minimal size, mature lymphocytes, ++ =medium size, ~5% immature lymphocytes, +++ =large size, ~10% immature lymphocytes, ++++ =extra-large size, ~20% immature lymphocytes.

f : − = none observed, + =several, ++ =readily apparent, +++ =cords filled.

g :− = absent, + =minimal foci.

h :− = absent, + = minimal foci.

i : = absent, + = minimal foci.

**Table 4:** Histology of the PLN of treatment groups in Experiment 1. Four days following sham injection or injection of vehicle controls, the indicated histological parameters were evaluated in the PLN.

**Parameter**	**Treatment Group**																	
	**Cr**_**2**_**O**_**3**_**0.0144E-3** **mg**		**Cr**_**2**_**O**_**3**_**0.0101** **mg**		**Cr**_**2**_**O**_**3**_**0.0216** **mg**		**Metal Salts 0.0998E-3** **mg**		**Metal Salts 0.0699** **mg**		**Metal Salts 0.150** **mg**		**Cr**_**2**_**O**_**3**_**+Metal Salts 0.114E-3** **mg**		**Cr**_**2**_**O**_**3**_**+Metal Salts 0.0800** **mg**		**Cr**_**2**_**O**_**3**_**+Metal Salts 0.171** **mg**	
	**Animal #**																	
	**489**	**490**	**499**	**500**	**379**	**380**	**509**	**510**	**369**	**370**	**359**	**360**	**329**	**330**	**339**	**340**	**349**	**350**
Number of secondary follicles/germinal centers^a^	++	−	−	−	−	+	**+**	**+**	−	−	+	−	−	−	−	−	−	++
Number of primary follicles^b^	+++	+++	++++	+++	++	+++	**+**	**++++**	+++	++	++	+	++	+	++	++	++	+++
Maturity of lymphocytes in cortex^c^	++	+	+	+	+	+	**+**	**+**	+	+	+	+	+	+	+	+	+	++
Lymphocyte hyperplasia in paracortex^d^	+	++	++	++	+	++	−	**++**	+	−	++	+	+	+	+	++	++	+++
Presence of plasma cells in medullary cords^e^	−	−	−	−	−	−	−	−	−	−	−	−	−	−	−	−	−	−
Necrosis^f^	−	−	−	−	−	−	−	−	−	−	−	−	−	−	−	−	−	−
Acute inflammation: Edema^g^	−	−	−	−	−	−	−	−	−	−	−	−	−	−	−	−	−	−
Acute inflammation: Polymorphonuclear cells^h^	−	−	−	−	−	−	−	−	−	+	−	−	−	−	−	−	−	−

a : − = absent, + =1–2 follicles, ++ =3–4 follicles.

b : − = no cortex present on sample, + =1–2 follicles, ++ =3–4 follicles, +++ =5–6 follicles, ++++ =7–8 follicles.

c : + =all small lymphocytes, ++ =~5% immature lymphocytes, +++ =~10% immature lymphocytes.

d : − = absent, + =minimal size, mature lymphocytes, ++ =medium size, ~5% immature lymphocytes, +++ =large size, ~10% immature lymphocytes, ++++ =extra-large size, ~20% immature lymphocytes.

e : −= none observed, + =several, ++ =readily apparent, +++ =cords filled.

f : − = absent, + =minimal foci.

g :− = absent, + = minimal foci.

h : − = absent, + = minimal foci.

**Table 5:** Reagents.

**Reagent**	**CAS #**	**Source**
Nickel chloride (NiCl_2_•6H_2_O)	7791-20-0	Sigma-Aldrich
Chromium chloride (CrCl_3_•6H_2_O)	10060-12-5	Sigma-Aldrich
Cobalt chloride (CoCl_2_•6H_2_O)	7791-13-1	Sigma-Aldrich
Chromium oxide particles (Cr_2_O_3_)	1308-38-9	Sigma-Aldrich
2,4-Dinitrochlorobenzene (DNCB)	97-00-7	Sigma-Aldrich
2,4-dichloronitrobenzene (DCNB)	611-06-3	Sigma-Aldrich
Sodium dodecyl sulfate (SDS)	151-21-3	Sigma-Aldrich
Bromodeoxyuridine (BrdU)	NA	Sigma-Aldrich
Dimethylsulfoxide (DMSO)	NA	Sigma-Aldrich
TiO_2_ particles (TiO_2_)	1317-70-0	US Research Nanomaterials, Inc
Potassium dichromate (K_2_Cr_2_O_7_)	7778-50-9	Fisher Scientific
Gold chloride (AuCl_3_)	13453-07-1	Acros Organics
Phosphate buffer saline (PBS)	NA	Hyclone
Syngeneic vehicle BALB/c mouse serum	NA	Charles River Laboratory
Flow cytometery antibodies	NA	BD Pharmingen or Acris Antibodies

**Table 6:** Treatment groups and doses used in Experiments 1 and 2.

	Treatment group	Vehicle	Dose (mg)	Number of mice for cellular endpoints per timepoint	Number of mice for histological endpoints.
***Experiment 1***					
Vehicle control	20% DMSO^a^	-	-	16	4
	Serum:PBS^b^	-	-	16	4
Chemical positive control	DNCB	20% DMSO	0.1250.3	88	22
Chemical negative control	SDS	20% DMSO	0.0938	8	2
	DCNB	20% DMSO	0.125	8	2
			0.3	8	2
Metal positive control	AuCl_3_	20% DMSO	0.0156	8	2
			0.0625	8	2
			0.125	8	2
	K_2_Cr_2_O_7_	20% DMSO	0.00625	8	2
			0.025	8	2
			0.050	8	2
Particle negative control	TiO_2_ particles	Serum:PBS	0.0210	8	2
Cr_2_O_3_ particles and/or metal salts^c^^,^^d^	Cr_2_O_3_ particles	Serum:PBS	0.0000144	8	2
			0.0101	8	2
			0.0216	8	2
	Metal Salts^c^	Serum:PBS	0.0000998	8	2
			0.0699	8	2
			0.150	8	2
	Cr_2_O_3_ particles + metal salts^c^^,^^d^	Serum:PBS	0.000114	8	2
			0.0800	8	2
			0.171	8	2
***Experiment 2***					
Vehicle control	20% DMSO^e^	-	-	5^e^	-
	Serum:PBS	-	-	10	-
Metal positive control	AuCl_3_	20% DMSO	0.125	10	-
	K_2_Cr_2_O_7_	20% DMSO	0.025	10	-
Cr_2_O_3_ particles and/or metal salts^c^^,^^d^	Cr_2_O_3_ particles	Serum:PBS	0.0216	10	-
	Cr_2_O_3_ particles + metal salts^c^^,^^d^	Serum:PBS	0.0005	10	-
			0.0025	10	-
			0.01	10	-
			0.04	10	-
			0.08	10	-

a In Experiment 1, there were two 20% DMSO groups (20% DMSO-A, 20% DMSO-B).

b In Experiment 1, there were two Serum:PBS groups (Serum:PBS-A, Serum:PBS-B).

c The ASTM F1537 standard specifications for wrought CoCr alloys used for surgical implants report that Co, Cr, and Ni content comprise approximately 64%, 28%, and ≤1% of the implant alloy, respectively. The ratio of total individual metal salts was based on these percentages, as discussed in [1].

d The ionic form of Co and Ni were administered. For Cr, a ratio of 62:38 particulate form (Cr_2_O_3_) to ionic form (Cr^3+^) was administered as discussed in [1].

e For the 20% DMSO group in Experiment 2, no mice were sacrificed at Day 4. At D7 and D11, 5 mice per group were sacrificed.
