# Supplementary material for: Data on the histological and immune cell response in the popliteal lymph node in mice following exposure to metal particles and ions
Source: Data Brief. 2016 Aug 27;9:388–97. doi: 10.1016/j.dib.2016.08.037 (PMC5035236; doi:10.1016/j.dib.2016.08.037)
Supplement: Supplementary file 2 — Supplementary material [file mmc2.zip › DIB S Figure 12 Footpad_V2.docx]

**Supplementary Figure 12:** Footpad swelling at 0 to 11 days following footpad injections in Experiment 2. Mice were injected with vehicle controls or positive controls (A) or test articles (B), as indicated. At the indicated day, the width of the footpad was measured. Data are presented as the mean ± SE. With the exception of 20% DMSO, the number of animals at each day for each treatment group was as follows: D0-D4: n = 30; D5-D7: n=20; D8-D11: n=10. For 20% DMSO, the number of animals at each day was as follows: D0-D7: n=10; D8-D11: n=5.

**A.**

**B.**
