# Supplementary material for: Data on the histological and immune cell response in the popliteal lymph node in mice following exposure to metal particles and ions
Source: Data Brief. 2016 Aug 27;9:388–97. doi: 10.1016/j.dib.2016.08.037 (PMC5035236; doi:10.1016/j.dib.2016.08.037)
Supplement: Supplementary file 2 — Supplementary material [file mmc2.zip › DIB S Figure 13 BW_V2.docx]

**Supplementary Figure 13:** Change in body weight at 4, 7 and 11 days after footpad injection in Experiment 2. Mice were weighed on D0 prior to injection with the indicated agents and again on the day of sacrifice (D4, D7 or D11). The % Initial BW is calculated by the following equation: BW_day of sacrifice_ / BW_D0_. Note that no D4 data were obtained for the 20% DMSO treatment group. The dashed line indicates a 10% loss in BW from D0, which is the threshold for clinically significant changes in BW. Data are presented as the mean ± SE.
