# Supplementary material for: Data on the histological and immune cell response in the popliteal lymph node in mice following exposure to metal particles and ions
Source: Data Brief. 2016 Aug 27;9:388–97. doi: 10.1016/j.dib.2016.08.037 (PMC5035236; doi:10.1016/j.dib.2016.08.037)
Supplement: Supplementary file 2 — Supplementary material [file mmc2.zip › DIB S Table 1 Inflam_V2.docx]

**Supplementary Table 1**: Clinical observations made throughout Experiment 1 regarding localized inflammation at injection site. Bold type indicates 50% or more of animals exhibited inflammation.

| **Group** | **Dose (mg)** | **Number of Animals with Localized Inflammation at Injection Site** | | | | |
| --- | --- | --- | --- | --- | --- | --- |
|  |  | **Hour 4** | **Day 1** | **Day 2** | **Day 3** | **Day 4** |
| *Vehicle controls* |  |  |  |  |  |  |
| 20% DMSO - A | 0 | 0/10 | 0/10 | 0/10 | 0/10 | 0/10 |
| 20% DMSO - B | 0 | 0/10 | 0/10 | 0/10 | 0/10 | 0/10 |
| Serum:PBS - A | 0 | 3/10 | 0/10 | 0/10 | 0/10 | 0/10 |
| Serum:PBS - B | 0 | **10/10** | 0/10 | 0/10 | 0/10 | 0/10 |
| Sham | 0 | 0/10 | 0/10 | 0/10 | 0/10 | 0/10 |
| *Chemical positive controls* |  |  |  |  |  |  |
| DNCB | 0.125 | **8/10** | **5/10** | 0/10 | 0/10 | 0/10 |
|  | 0.3 | **10/10** | **8/10** | **8/10** | **6/10** | 4/10 |
| *Chemical negative control* |  |  |  |  |  |  |
| SDS | 0.0938 | **6/10** | **7/10** | **7/10** | 2/10 | 1/10 |
| DCNB | 0.125 | **6/10** | 0/10 | 0/10 | 0/10 | 0/10 |
|  | 0.3 | **7/10** | 0/10 | 0/10 | 0/10 | 0/10 |
| *Metal positive controls* |  |  |  |  |  |  |
| AuCl_3_ | 0.0156 | **10/10** | 3/10 | 0/10 | 0/10 | 0/10 |
|  | 0.0625 | **10/10^a^** | **10/10** | **10/10** | **10/10** | **10/10** |
|  | 0.125 | **10/10** | **10/10** | **10/10** | **10/10** | **10/10** |
| K_2_Cr_2_O_7_ | 0.00625 | 0/10 | 0/10 | 0/10 | 0/10 | 0/10 |
|  | 0.025 | 1/10 | 0/10 | 0/10 | 0/10 | 1/10 |
|  | 0.050 | 1/10 | 1/10 | 1/10 | 0/10 | **7/10** |
| *Metal negative control* |  |  |  |  |  |  |
| TiO_2_ particles | 0.0210 | 10/10 | 0/10 | 0/10 | 0/10 | 0/10 |
| *Cr_2_O_3_ particles and/or metal salts* | |  |  |  |  |  |
| Cr_2_O_3_ particles | 0.0000144 | **10/10** | 0/10 | 0/10 | 0/10 | 0/10 |
|  | 0.0101 | **10/10** | 0/10 | 0/10 | 0/10 | 0/10 |
|  | 0.0216 | **9/10** | 0/10 | 0/10 | 0/10 | 0/10 |
| Metal salts | 0.0000998 | **10/10** | 0/10 | 0/10 | 0/10 | 0/10 |
|  | 0.0699 | **5/10** | 0/10 | 0/10 | 0/10 | 0/10 |
|  | 0.150 | **9/10** | **5/10** | **5/10** | 3/10 | 0/10 |
| Cr_2_O_3_ particles + metal salts | 0.000114 | 3/10 | 0/10 | 0/10 | 0/10 | 0/10 |
|  | 0.0800 | **10/10** | 2/10 | 2/10 | 0/10 | 0/10 |
|  | 0.171 | **10/10** | **5/10** | **5/10** | 0/10 | 0/10 |

^a^ Animals held dosed foot up when walking.
