# Supplementary material for: Data on the histological and immune cell response in the popliteal lymph node in mice following exposure to metal particles and ions
Source: Data Brief. 2016 Aug 27;9:388–97. doi: 10.1016/j.dib.2016.08.037 (PMC5035236; doi:10.1016/j.dib.2016.08.037)
Supplement: Supplementary file 2 — Supplementary material [file mmc2.zip › DIB S Table 2 Redness_V2.docx]

**Supplementary Table 2***:* Clinical observations made throughout Experiment 1 regarding discoloration as noted in footnotes. Bold type indicates 50% or more of animals exhibited discoloration.

| **Group** | **Dose (mg)** |  | **Number of Animals with Discoloration at Dose Site or Foot** | | | | |
| --- | --- | --- | --- | --- | --- | --- | --- |
|  |  | **n** | **Hour 4** | **Day 1** | **Day 2** | **Day 3** | **Day 4** |
| *Vehicle controls* |  |  |  |  |  |  |  |
| 20% DMSO - A | 0 | 10 | 0/10 | 0/10 | 0/10 | 0/10 | 0/10 |
| 20% DMSO - B | 0 | 10 | 1/10^a^ | 1/10^a^ | 0/10 | 0/10 | 0/10 |
| Serum:PBS - A | 0 | 10 | 0/10 | 0/10 | 0/10 | 0/10 | 0/10 |
| Serum:PBS - B | 0 | 10 | 0/10 | 0/10 | 0/10 | 0/10 | 0/10 |
| Sham | 0 | 10 | 0/10 | 0/10 | 0/10 | 0/10 | 0/10 |
| *Chemical positive controls* |  |  |  |  |  |  |  |
| DNCB | 0.125 | 10 | 0/10 | 0/10 | 0/10 | 0/10 | 0/10 |
|  | 0.3 | 10 | 0/10 | 1/10^a^ | 1/10^a^ | 0/10 | 0/10 |
| *Chemical negative control* |  |  |  |  |  |  |  |
| SDS | 0.0938 | 10 | 0/10 | 0/10 | 0/10 | 0/10 | 0/10 |
| DCNB | 0.125 | 10 | 0/10 | 0/10 | 0/10 | 0/10 | 0/10 |
|  | 0.3 | 10 | 0/10 | 0/10 | 0/10 | 0/10 | 0/10 |
| *Metal positive controls* |  |  |  |  |  |  |  |
| AuCl_3_ | 0.0156 | 10 | 0/10 | 0/10 | 0/10 | 0/10 | 0/10 |
|  | 0.0625 | 10 | 0/10 | 0/10 | 0/10 | 0/10 | 0/10 |
|  | 0.125 | 10 | 0/10 | 0/10 | 0/10 | 0/10 | 0/10 |
| K_2_Cr_2_O_7_ | 0.00625 | 10 | 0/10 | 0/10 | 0/10 | 0/10 | 0/10 |
|  | 0.025 | 10 | 1/10^b^ | 1/10^b^ | 1/10^b^ | 0/10 | 0/10 |
|  | 0.050 | 10 | 0/10 | 0/10 | 0/10 | 0/10 | 0/10 |
| *Metal negative control* |  |  |  |  |  |  |  |
| TiO_2_ particles | 0.0210 | 10 | 0/10 | 0/10 | 0/10 | 0/10 | 0/10 |
| *Cr_2_O_3_ particles and/or metal salts* | |  |  |  |  |  |  |
| Cr_2_O_3_ particles | 0.0000144 | 10 | 0/10 | 0/10 | 0/10 | 0/10 | 0/10 |
|  | 0.0101 | 10 | 0/10 | 0/10 | 0/10 | 0/10 | 0/10 |
|  | 0.0216 | 10 | 0/10 | 3/10^a^ | 3/10^a^ | 0/10 | 0/10 |
| Metal salts | 0.0000998 | 10 | 0/10 | 0/10 | 0/10 | 0/10 | 0/10 |
|  | 0.0699 | 10 | 0/10 | 0/10 | 0/10 | 0/10 | 0/10 |
|  | 0.150 | 10 | 0/10 | 2/10^c^ | 2/10^c^ | 0/10 | 0/10 |
| Cr_2_O_3_ particles + metal salts | 0.000114 | 10 | 0/10 | 0/10 | 0/10 | 0/10 | 0/10 |
|  | 0.0800 | 10 | 0/10 | 0/10 | 0/10 | 0/10 | 0/10 |
|  | 0.171 | 10 | 0/10 | **6/10^d^** | **6/10^d^** | **6/10^d^** | 2/10^e^ |

^a^ Dose site discolored dark.

^b^ Discolored on middle digit and top of foot.

^c^ Foot dark red.

^d^ Four animals with dose site discolored dark and two animals with foot dark red.

^e^ One animal with dose site discolored dark and one animal with foot dark red.
