# Supplementary material for: Data on the histological and immune cell response in the popliteal lymph node in mice following exposure to metal particles and ions
Source: Data Brief. 2016 Aug 27;9:388–97. doi: 10.1016/j.dib.2016.08.037 (PMC5035236; doi:10.1016/j.dib.2016.08.037)
Supplement: Supplementary file 2 — Supplementary material [file mmc2.zip › DIB S Table 4 Redness_V2.docx]

**Supplementary Table 4**: Clinical observations made throughout Experiment 2 regarding discoloration at injection site. Bold type indicates 50% or more of animals exhibited discoloration.

| **Group** | **Dose (mg)** | **Number of Animals with Redness at Injection Site** | | | | | | | | | | | | | |
| --- | --- | --- | --- | --- | --- | --- | --- | --- | --- | --- | --- | --- | --- | --- | --- |
|  |  | **Injection** | **Hour 1** | **Hour 4** | **Day 1** | **Day 2** | **Day 3** | **Day 4** | **Day 5** | **Day 6** | **Day 7** | **Day 8** | **Day 9** | **Day 10** | **Day 11** |
| *Vehicle controls* |  |  |  |  |  |  |  |  |  |  |  |  |  |  |  |
| 20% DMSO | 0 | 0/10 | 0/10 | 0/10 | 0/10 | 0/10 | 0/10 | 0/10 | 0/10 | 0/10 | 0/10 | 0/5 | 0/5 | 0/5 | 0/5 |
| Serum:PBS | 0 | 0/30 | **30/30** | 0/30 | 0/30 | 0/30 | 0/30 | 0/30 | 0/20 | 0/20 | 0/20 | 0/10 | 0/10 | 0/10 | 0/10 |
| *Metal positive controls* |  |  |  |  |  |  |  |  |  |  |  |  |  |  |  |
| AuCl_3_ | 0.125 | 0/30 | **30/30** | **30/30** | **30/30** | 0/30 | 0/30 | 0/30 | 0/20 | 0/20 | 0/20 | 0/10 | 0/10 | 0/10 | 0/10 |
| K_2_Cr_2_O_7_ | 0.025 | 0/30 | **30/30** | **30/30** | **30/30** | 0/30 | 0/30 | 0/30 | 0/20 | 0/20 | 0/20 | 0/10 | 0/10 | 0/10 | 0/10 |
| *Cr_2_O_3_ particles ± metal salts* | |  |  |  |  |  |  |  |  |  |  |  |  |  |  |
| Cr_2_O_3_ particles | 0.0216 | 0/30 | **30/30** | 0/30 | 0/30 | 0/30 | 0/30 | 0/30 | 0/20 | 0/20 | 0/20 | 0/10 | 0/10 | 0/10 | 0/10 |
| Cr_2_O_3_ particles + metal salts | 0.0005 | 0/30 | **30/30** | 0/30 | 0/30 | 0/30 | 0/30 | 0/30 | 0/20 | 0/20 | 0/20 | 0/10 | 0/10 | 0/10 | 0/10 |
|  | 0.0025 | 0/30 | **30/30** | 0/30 | 0/30 | 0/30 | 0/30 | 0/30 | 0/20 | 0/20 | 0/20 | 0/10 | 0/10 | 0/10 | 0/10 |
|  | 0.01 | 0/30 | **30/30** | 0/30 | 0/30 | 0/30 | 0/30 | 0/30 | 0/20 | 0/20 | 0/20 | 0/10 | 0/10 | 0/10 | 0/10 |
|  | 0.04 | 0/30 | **28/30** | 0/30 | 0/30 | 0/30 | 0/30 | 0/30 | 0/20 | 0/20 | 0/20 | 0/10 | 0/10 | 0/10 | 0/10 |
|  | 0.08 | 0/30 | **30/30** | **30/30** | 0/30 | 0/30 | 0/30 | 0/30 | 0/20 | 0/20 | 0/20 | 0/10 | 0/10 | 0/10 | 0/10 |
