# Supplementary material for: Data on the histological and immune cell response in the popliteal lymph node in mice following exposure to metal particles and ions
Source: Data Brief. 2016 Aug 27;9:388–97. doi: 10.1016/j.dib.2016.08.037 (PMC5035236; doi:10.1016/j.dib.2016.08.037)
Supplement: Supplementary file 2 — Supplementary material [file mmc2.zip › DIB S Figure 2 Footpad_V2.docx]

**Supplementary Figure 2:** Footpad swelling at 1, 2 and 4 days following footpad injection in Experiment 1. Mice were sham injected, or injected with the indicated test articles. On D1, D2 and D4 post injection, the width of the footpad was measured. Data are presented as the mean ± SE.
