# Supplementary material for: Data on the histological and immune cell response in the popliteal lymph node in mice following exposure to metal particles and ions
Source: Data Brief. 2016 Aug 27;9:388–97. doi: 10.1016/j.dib.2016.08.037 (PMC5035236; doi:10.1016/j.dib.2016.08.037)
Supplement: Supplementary file 2 — Supplementary material [file mmc2.zip › DIB S Figure 3 BW_V2.docx]

**Supplementary Figure 3:** Change in body weight 4 days after footpad injection in Experiment 1. Mice were weighed on D0 prior to sham injection or injection with the indicated agents and again on D4 at sacrifice. The % Initial BW is calculated by the following equation: BW_D4_ / BW_D0_. The dashed line indicates a 10% loss in BW from D0, which is the threshold for clinically significant changes in BW. Data are presented as the mean ± SE.
