# Supplementary material for: Data on the histological and immune cell response in the popliteal lymph node in mice following exposure to metal particles and ions
Source: Data Brief. 2016 Aug 27;9:388–97. doi: 10.1016/j.dib.2016.08.037 (PMC5035236; doi:10.1016/j.dib.2016.08.037)
Supplement: Supplementary file 2 — Supplementary material [file mmc2.zip › DIB S Figure 5 FC CD3B220_V2.docx]

**Supplementary Figure 5:** Representative dot plots depicting the percentage of CD3^+^ and B220^+^ cells in the PLN of mice four days after receiving a footpad injection of Cr_2_O_3_ particles, metal salts, or Cr_2_O_3_ particles + metal salts in Experiment 1. The number represents the percentage of CD3^+^ and B220^+^ cells in the representative samples. Flow cytometry data were collected on separate days, and the data from each day are presented in **A** and **B**.

**A.**

**
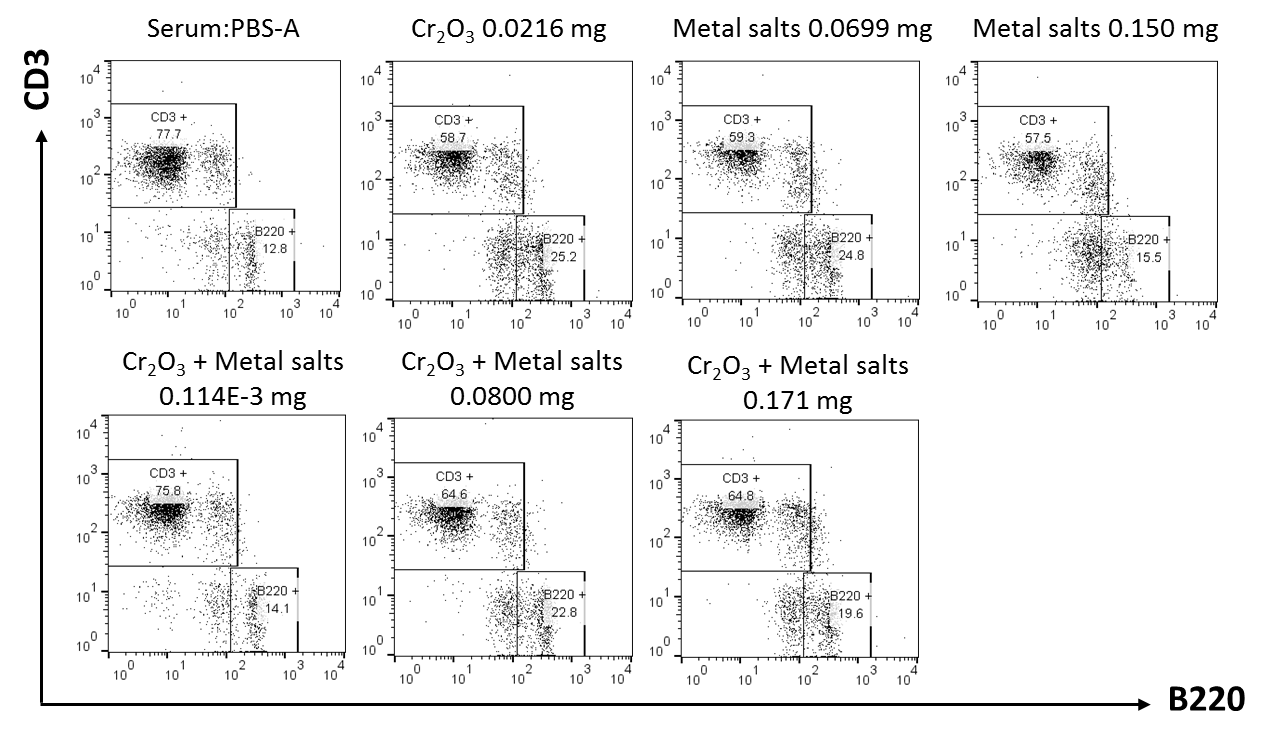
**

**B.**

**
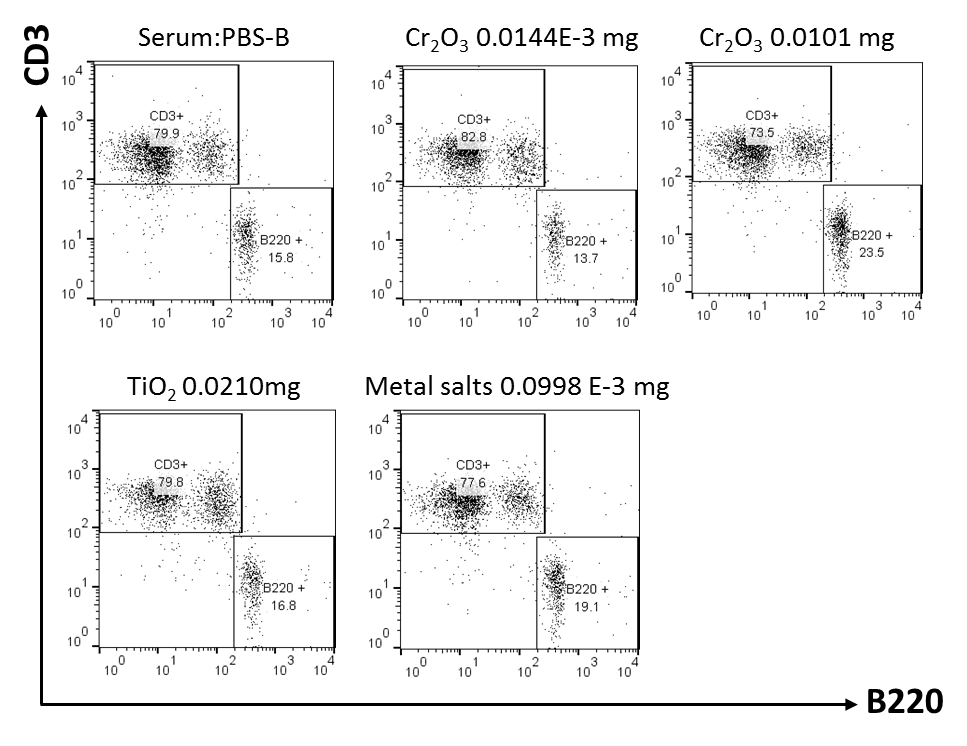
**
