# Supplementary figures and images for: Data on the histological and immune cell response in the popliteal lymph node in mice following exposure to metal particles and ions
Source: Data Brief. 2016 Aug 27;9:388–97. doi: 10.1016/j.dib.2016.08.037 (PMC5035236; doi:10.1016/j.dib.2016.08.037)

**Supplementary Figure 1**: SEM image (A) and chemical composition (B) of TiO2 particles.

**A.**


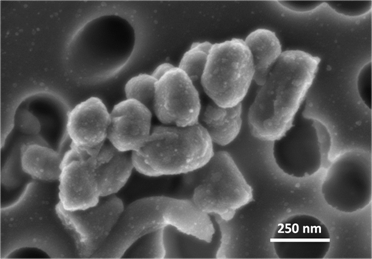


**B.**


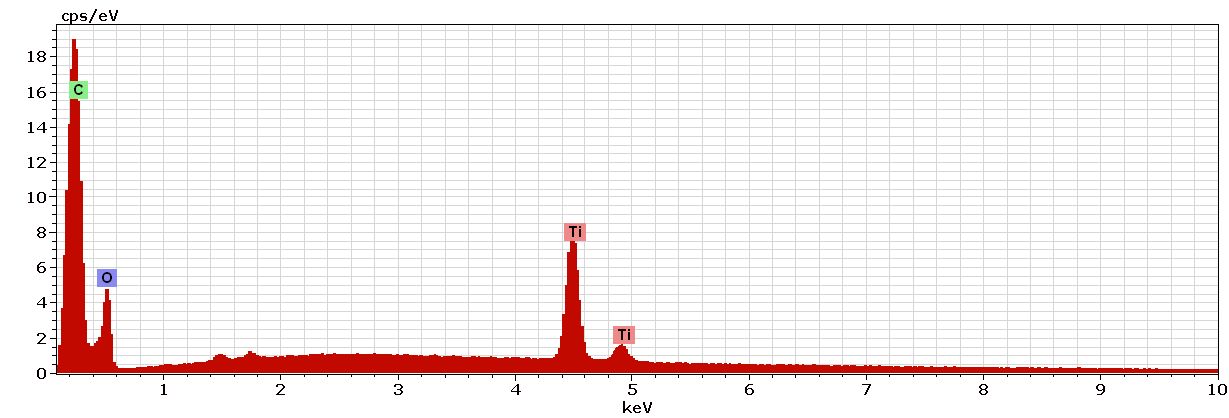

Supplement: Supplementary file 2 — Supplementary material [file mmc2.zip › DIB S Figure 1 TiO2_V2.docx]
